# CXCR3 May Help Regulate the Inflammatory Response in Acute Lung Injury via a Pathway Modulated by IL-10 Secreted by CD8 + CD122+ Regulatory T Cells

**DOI:** 10.1007/s10753-015-0276-0

**Published:** 2015-10-16

**Authors:** Li Nie, Wei Wu, Zhibing Lu, Gangyan Zhu, Juan Liu

**Affiliations:** Department of Geriatrics, Renmin Hospital of Wuhan University, 238, Jiefang Road, Wucang, Wuhan, 430060 China; Clinical Laboratory Department, Renmin Hospital of Wuhan University, Wuhan, China; Department of Cardiology, Renmin Hospital of Wuhan University, Wuhan, China

**Keywords:** acute lung injury, CXCR3, CD8 + CD122+ regulatory T cells, interleukin-10

## Abstract

The aim of this study is to investigate the role of CXCR3 and IL-10 in lipopolysaccharide (LPS)-induced acute lung injury (ALI). ALI was induced by LPS injection (10 mg/kg) via the tail vein in C57BL/6 mice. Mice were sacrificed after 2 or 12 h to examine the levels of inflammatory cytokines in bronchoalveolar lavage fluid (BALF) and histopathologic assessments. At 12 h after LPS injection, mice exhibited more severe lung infiltration by CD8+ T cell and less infiltration by CD8+CD122+ regulatory T cells than at 2 h after LPS challenge or in the control (mice not exposed to LPS). At 12 h, IFN-γ, CXCR3, and CXCL10 were significantly higher in the lungs. IL-10 in the lungs was significantly lower. CXCR3 may help to recruit CD8+ T cells and promotes IFN-γ and CXCL10 release. Such effects could be inhibited by IL-10 secreted by CD8+CD122+ regulatory T cells.

## INTRODUCTION

Acute lung injury (ALI) and acute respiratory distress syndrome (ARDS) are frequent complications in patients with burns, trauma, and sepsis, and they are associated with high rates of morbidity and mortality [1]. Infection or mechanical injury triggers a generalized inflammatory response involving various inflammatory cells and pro-inflammatory mediators. In particular, several studies suggest that the chemokine receptor CXCR3, which is expressed primarily on activated T lymphocytes, natural killer (NK) cells and some epithelial cells, can trigger an inflammatory response cascade at sites of ALI by recruiting CD8+ T cells and promoting release of pro-inflammatory interferon-γ (IFN-γ) and chemokine CXCL10 [2].

The body is capable of mitigating the inflammation in ALI by releasing anti-inflammatory mediators at injury sites, leading investigators to examine how the disbalance between pro- and anti-inflammatory factors may drive ALI and may be manipulated for therapeutic effects [3–5]. For example, interleukin-10 (IL-10) secreted by CD8+CD122+ regulatory T cells has been shown to inhibit CD8+ T cells and reduce inflammation in various inflammatory diseases [6]. However, whether CD8+CD122+ regulatory T cells and IL-10 regulate CXCR3 activity in ALI has not been reported.

In the current study, we examined whether CXCR3 could interact with IL-10 secreted by CD8+CD122+ regulatory T cells in a mouse model of ALI induced by lipopolysaccharide (LPS) exposure.

## MATERIAL AND METHODS

### Mouse Model of ALI

Healthy male C57BL/6 mice aged 10–12 weeks and weighing 20–22 g (SJA Laboratory Animals, Hunan, China)were randomly divided into a control group (*n* = 8) and two ALI groups (*n* = 8 each). At baseline, control mice were injected in the tail vein with 1 ml saline, while ALI mice were injected with 10 mg/kg lipopolysaccharide (Sigma, Beijing, China) dissolved in 1 ml saline. One group of ALI mice was sacrificed by pentobarbital overdose at 2 h after injection, while the other ALI group as well as the control group was sacrificed at 12 h after injection.

All experiments were conducted according to international and institutional guidelines for animal care, and the study protocol was approved by the Research Ethics Committee of Renmin Hospital, Wuhan University.

### Lung Tissue Histology

The lower lobe of the left lung was removed, inflated to 250 mmH_2_O with 10 % formal in, fixed overnight, embedded in paraffin, and sectioned to a thickness of 5 μm. Sagittal sections were stained with hematoxylin-eosin (HE) for histopathologic assessments under a light microscope (Olympus, Japan) by an experience pathologist blinded to treatment conditions.

### Harvesting of Bronchoalveolar Lavage Fluid

Tracheas of sacrificed animals were intubated with a 20-gauge catheter. Bronchoalveolar lavage fluid (BALF) was collected by flushing twice with 0.8 mL of ice-cold phosphate-buffered saline (PBS). A total injected volume of 1.5 mL was recovered in >95 % of mice. The recovered BALF was centrifuged at 1500 rpm for 5 min at 4 °C, and the supernatant was stored at −70 °C for subsequent assay of cytokines and chemokines. Total cells were counted on a hemocytometer. For differential cell counting, cells were spun onto glass slides, fixed, and stained with Diff-Quik reagents (Beijing Chemical Works, Beijing, China). Numbers of macrophages, neutrophils, and lymphocytes per 400 cells were determined based on morphology.

### Antibody Labeling of Cells from BALF

BALF cells (2 × 10^7^/mL) resuspended in 50 μL PBS were incubated for 15 min on ice with 10-μL blocking buffer (1 μL blocking antibody Fc +9 μL PBS containing 2 % bovine serum albumin (Sigma)). Cells were washed once with PBS and then incubated for 1 h on ice with 50 μL FITC-conjugated anti-CD122 antibody (Sigma), PE-conjugated anti-CD8 antibody (Sigma), or control mouse IgG2b (Sigma). Cells were washed twice with PBS, then fixed in PBS containing 2 % formalin. Cells were sorted by flow cytometry on a FACScan cytometer (BD, Franklin L, New Jersey, USA).

### Determination of Cytokine and Chemokine Levels in BALF Using Enzyme-Linked Immunosorbent Assay (ELISA)

Commercial ELISA kits (R&D Systems, Minneapolis, MN, USA) were used to measure concentrations of IFN-γ, CXCR3, CXCL10, and IL-10 in BALF.

### Quantitation of Cytokine and Chemokine mRNA Levels in Lung Tissue

Total cellular RNA was isolated using TRIzol reagent (Invitrogen, Carlsbad, CA, USA) from sections of lung tissue weighing 100 mg. Total RNA was reverse-transcribed using the Reverse Transcription Kit (Invitrogen), and the resulting cDNA was subjected to PCR to measure levels of mRNAs encoding IFN-γ, CXCR3, CXCL10, or IL-10 (Table 1). Transcript levels were quantified using the △*C*_T_ method, in which *C*_T_ refers to the threshold number of cycles and 2^−△△CT^ refers to the amount of target transcript relative to the endogenous control β-actin.

**Table 1:** PCR Primers and Annealing Temperatures for Quantitation of Cytokine and Chemokine Transcript Levels

Target	F/R	Sequence(5′ to 3′)	Tm (°C)	Product (bp)
IFN-γ	F	CCCCGCAGTATTGATGAGTT	56	194
	R	TTGGAATAGTTGCCCGAGTC		
CXCR3	F	ACTACGATCAGCGCCTCAAT	56	163
	R	CCTCTGGAGACCAGCAGAAC		
CXCL10	F	AAGTGCTGCCGTCATTTTCT	56	186
	R	GTGGCAATGATCTCAACACG		
IL-10	F	CCAAGCCTTATCGGAAATGA	56	162
	R	TTTTCACAGGGGAGAAATCG		
β-actin	F	CACGATGGAGGGGCCGGACTCATC	56	240
	R	TAAAGACCTCTATGCCAACACAGT		

*F* forward, *R* reverse

### Statistical Analysis

Data are expressed as mean ± SD. Comparisons between two groups were carried out using the non-parametric two-tailed *t* test. Possible correlations between different factors were explored across all three groups using Pearson correlation analysis. All analyses were performed using SPSS19.0 (IBM, Chicago, IL, USA). *P* < 0.05 was considered statistically significant.

## RESULTS

### Lung Histopathology in LPS-Induced ALI

**Fig. 1:**
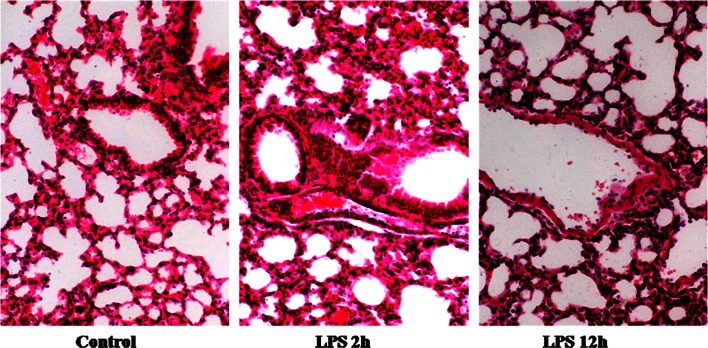
Representative photomicrographs of lung tissues stained with hematoxylin-eosin from control mice and mice with acute lung injury harvested at 2 and 12 h after lipopolysaccharide (LPS) injection. Magnification, ×400.

Lung tissue from control mice appeared normal with no significant signs of inflammation or of inflammatory cells in the pulmonary alveolus (Fig. 1). Tissue sections from ALI mice showed much greater accumulation of leukocytes in the pulmonary parenchyma and peribronchus at 12 h than at 2 h or in mice not exposed to LPS.

### Infiltration of Inflammatory Cells into the Airways and Lungs

To determine whether LPS affects the infiltration of inflammatory cells into airways, we measured the sizes of different T cell populations in BALF. Populations of various inflammatory cells, including leukocytes, lymphocytes, neutrophils, and macrophages were larger in BALF from ALI mice at 12 h than in BALF from the other two groups, indicating a stronger inflammatory response. Populations of leukocytes and neutrophils were significantly higher in BALF from ALI mice at 2 h than in BALF from control mice (Table 2).

**Table 2:** Inflammatory Cell Counts in Bronchoalveolar Lavage Fluid from Control Mice and Mice with Lipopolysaccharide-Induced Acute Lung Injury (ALI) after 2 or 12 h

Cell type	Control (*n* = 8)	ALI at 2 h (*n* = 8)	ALI at 12 h (*n* = 8)
Leukocytes, ×10^5^	2.03 ± 0.51	5.88 ± 0.89*^#^	39.90 ± 4.00*
Lymphocytes, ×10^3^	0.33 ± 0.01	0.34 ± 0.01^#^	0.36 ± 0.01*
Neutrophils, ×10^2^	0.14 ± 0.03	0.29 ± 0.03*^#^	0.43 ± 0.05*
Macrophages, ×10^2^	0.82 ± 0.06	0.83 ± 0.05^#^	0.99 ± 0.06*

Results are expressed as mean ± SD

*P < 0.05 vs control group; ^#^ *P* < 0.05 vs ALI at 12 h

### Populations of CD8+ T Cells and CD8+CD122+ T Cells in Airways and Lung Tissue

**Fig. 2:**
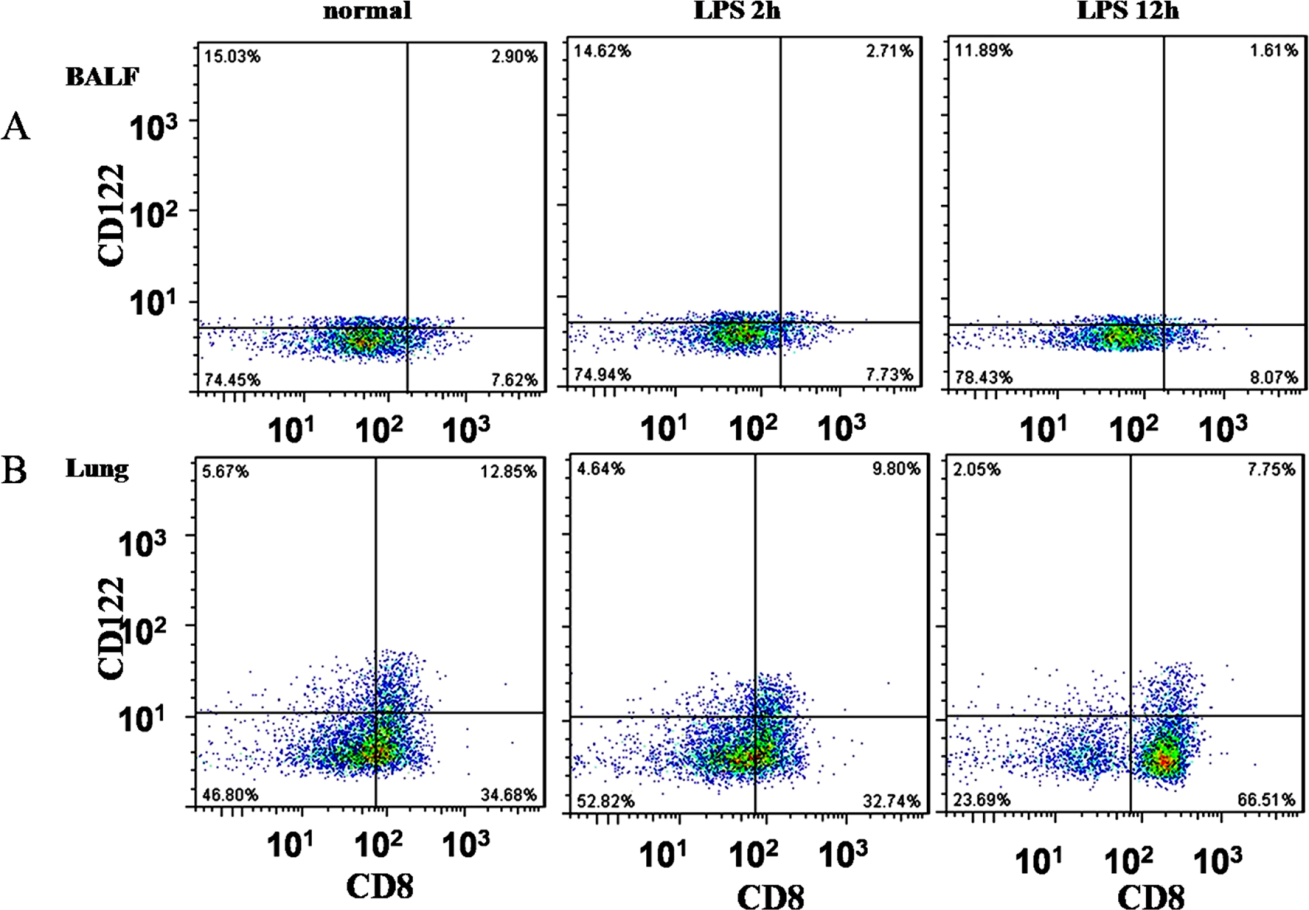
Infiltration of the **a** airways and **b** lungs by CD8+ T cells and CD8+CD122+ T cells in control mice and mice with acute lung injury at 2 and 12 h after lipopolysaccharide (LPS) injection.

The percentage of CD8+ T cells in both BALF and lung tissue was significantly higher in ALI mice at 12 h than in either control mice or ALI mice at 2 h. Conversely, the percentage of CD8+CD122+ regulatory T cells was lowest in ALI mice at 12 h, higher in ALI mice at 2 h, and highest in control animals (Fig. 2, Table 3). Percentages of CD8 + CD122+ regulatory T cells correlated negatively with percentages of CD8+ T cells in both BALF (*r* = −0.797) and lungs (*r* = −0.994).

**Table 3:** Percentages of CD8+ and CD8 + CD122+ T cells in Bronchoalveolar Lavage Fluid and Lung Tissue from Control Mice and Mice with Lipopolysaccharide-Induced Acute Lung Injury (ALI) After 2 or 12 h

Cell type	Control (*n* = 8)	ALI at 2 h (*n* = 8)	ALI at 12 h (*n* = 8)
Bronchoalveolar lavage fluid			
CD8+ T cells, %	7.61 ± 0.11	7.70 ± 0.15^#^	8.07 ± 0.17*
CD8+CD122+T cells, %	15.05 ± 0.21	14.61 ± 0.14*^#^	11.94 ± 0.13*
Lung tissue			
CD8+ T cells, %	34.65 ± 0.13	32.72 ± 0.13^#^	66.53 ± 0.19*
CD8+CD122+T cells, %	5.67 ± 0.09	4.63 ± 0.09*^#^	2.05 ± 0.08*

Results are expressed as mean ± SD

*P < 0.05 vs control group; ^#^ *P* < 0.05 vs ALI at 12 h

### Levels of Inflammatory Cytokines and CXCL10 in BALF

**Fig. 3:**
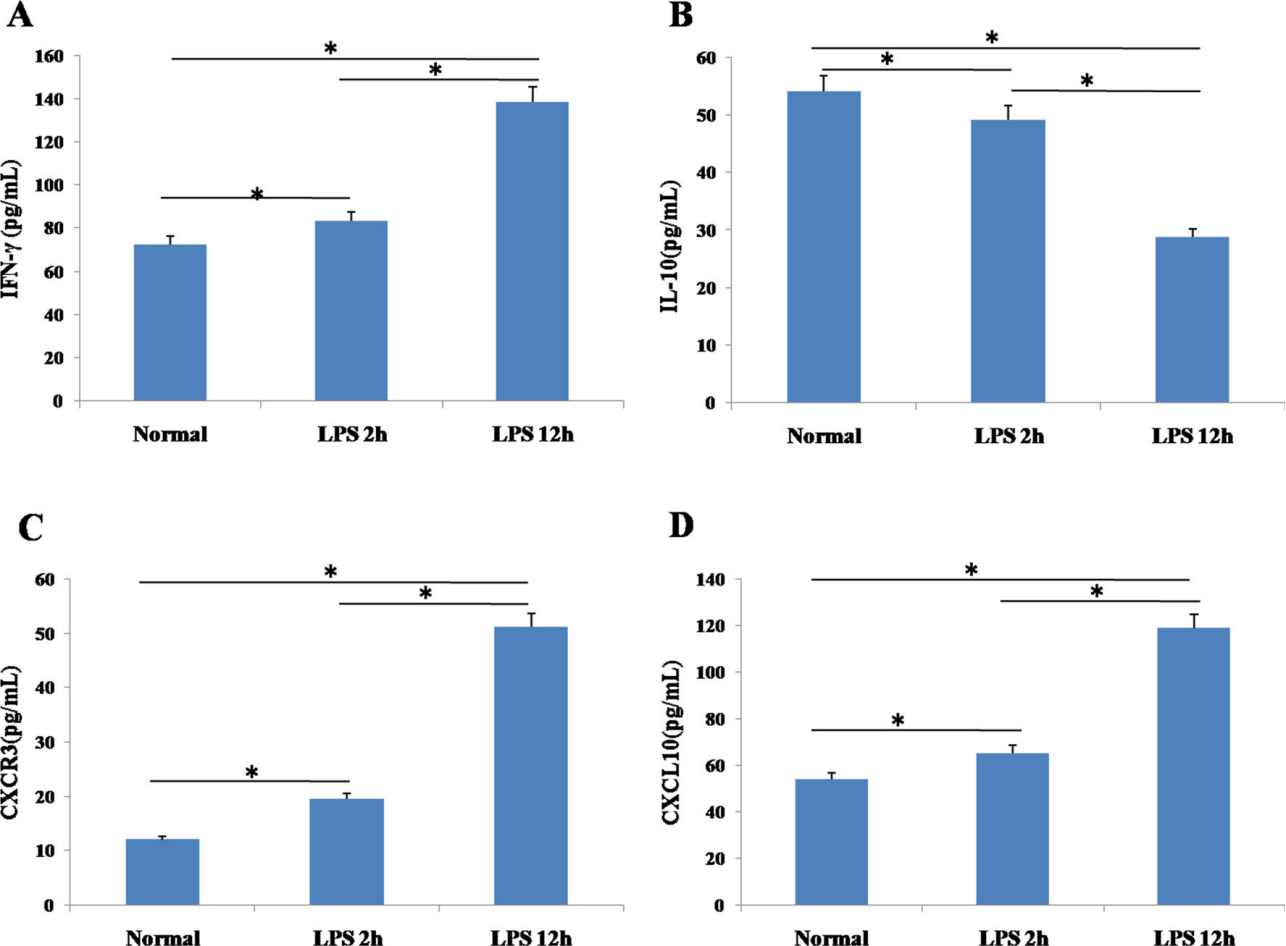
ELISA determination of **a** IFN-γ, **b** IL-10, **c** CXCR3, and **d** CXCL10 in bronchoalveolar lavage fluid from control mice and mice with acute lung injury at 2 and 12 h after lipopolysaccharide (LPS) injection (*n* = 8 per group). Results are expressed as mean ± SD. *P < 0.05.

BALF from ALI mice at 2 h contained significantly higher protein levels of CXCR3, CXCL10, and IFN-γ than BALF from the control group, and the levels of all three proteins were significantly higher at 12 h than at 2 h (Fig. 3, Table 4). Conversely, IL-10 concentration was significantly lower in BALF from ALI mice at 12 h than in ALI mice at 2 h; the concentration was in turn significantly lower in ALI mice at 2 h than in control animals. Levels of CXCR3 protein correlated negatively with levels of IL-10 protein in BALF (*r* = −0.969).

**Table 4:** Concentrations of Inflammatory Cytokines and CXCL10 in Bronchoalveolar Lavage Fluid from Control Mice and Mice with Lipopolysaccharide-Induced Acute Lung Injury (ALI) After 2 or 12 h

Factor	Concentration (pg/mL)		
	Control (*n* = 8)	ALI at 2 h (*n* = 8)	ALI at 12 h (*n* = 8)
IFN-γ	72.59 ± 4.13	83.42 ± 4.71*^#^	138.43 ± 9.42*
IL-10	54.17 ± 3.24	49.26 ± 2.82*^#^	28.77 ± 2.05*
CXCR3	11.99 ± 1.13	19.53 ± 1.62*^#^	51.13 ± 2.42*
CXCL10	53.91 ± 4.31	65.18 ± 5.88*^#^	118.75 ± 7.18*

Results are expressed as mean ± SD

*P < 0.05 vs control group; ^#^ *P* < 0.05 vs ALI at 12 h

### Levels of Cytokine and Chemokine mRNA in Lungs

**Fig. 4:**
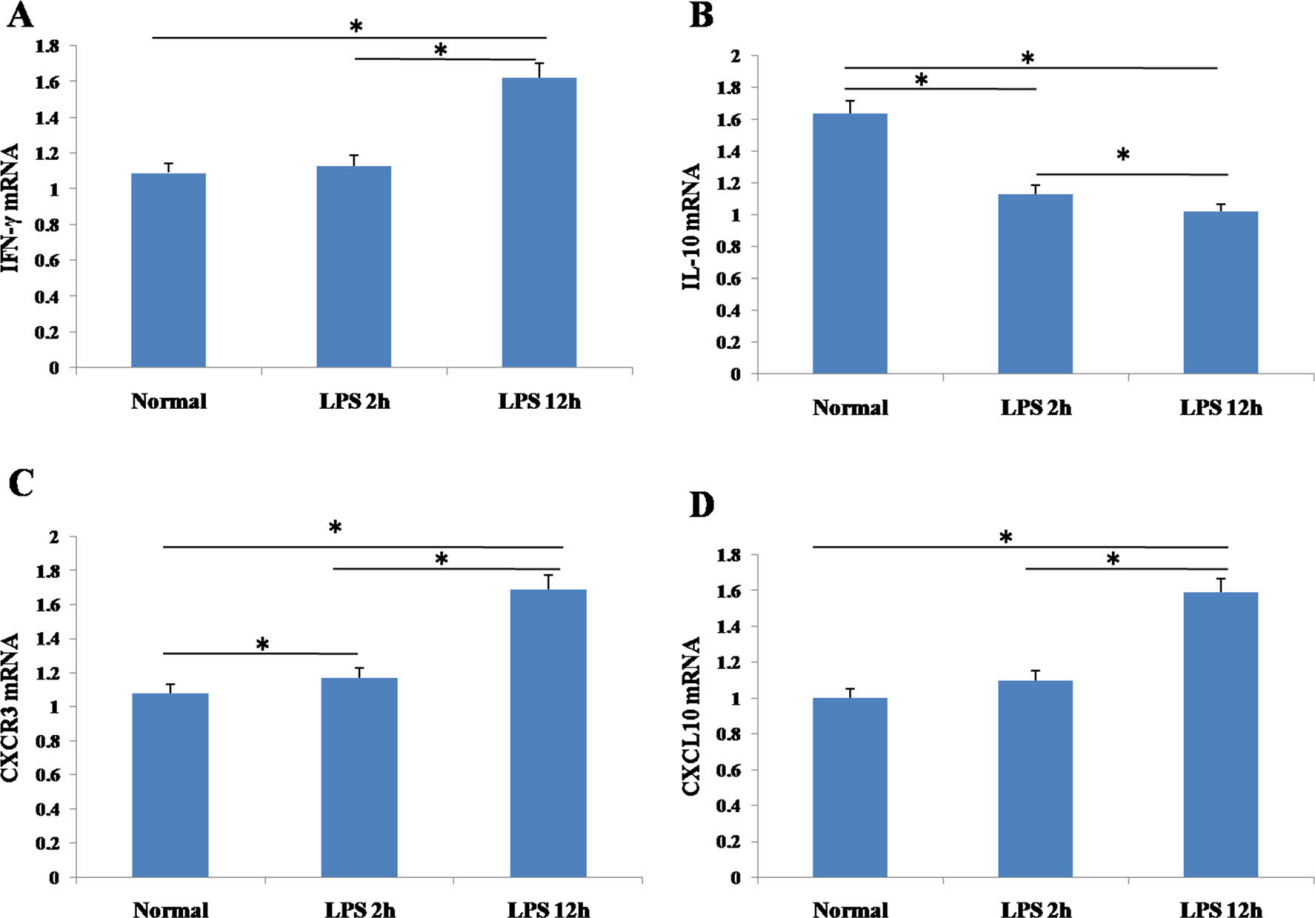
RT-PCR quantitation of mRNA levels of **a** IFN-γ, **b** IL-10, **c** CXCR3, and **d** CXCL10 in lung tissues from control mice and mice with acute lung injury at 2 and 12 h after lipopolysaccharide (LPS) injection (*n* = 8 per group). Results are expressed as mean ± SD. *P < 0.05.

Consistent with our analyses of protein levels in BALF, levels of mRNA encoding CXCR3, CXCL10 and IFN-γ were markedly higher in lungs of ALI mice at 12 h than at 2 h or in mice not exposed to LPS (Fig. 4). Levels of CXCR3 mRNA were significantly higher in ALI mice at 2 h than in healthy animals. Also consistent with the BALF analyses, levels of IL-10 mRNA were highest in the control group, lower in the ALI mice at 2 h, and lowest in the ALI mice at 12 h. Levels of CXCR3 mRNA correlated negatively with levels of IL-10 mRNA in lung tissue (*r* = −0.664).

## DISCUSSION

Using a mouse model of LPS-induced ALI, we show that injury progression is associated with decreased expression and secretion of IL-10 and increased expression of CXCR3, CXCL10, and IFN-γ. At the same time, progression is associated with an increase in CD8+ T cells and a decrease in CD8+CD122+ regulatory T cells in both BALF and lung tissue. These results are consistent with the known pro-inflammatory effects of CXCR3 and the known ability of CD8+CD122+ regulatory T cells to reduce inflammation by secreting IL-10. Taken together, our results provide preliminary evidence that restoring a balance between CXCR3 and IL-10 may help prevent or treat ALI.

CXCR3, a receptor for the inflammatory chemokines CXCL9, CXCL10, and CXCL11, is preferentially expressed on activated CD8+ T cells as well as Th1 cells and is thought to play a critical role in trafficking to sites of inflammation and in mediating biological functions such as cell migration and proliferation [7, 8]. CXCR3 and its ligands are involved in the pathogenesis of infection, autoimmune diseases, acute allograft rejection, and tumor growth [7, 9, 10]. During infection, injury, or immuno-inflammatory response, IFN-γ strongly induces the expression of CXCR3 and CXCL10 primarily on activated T cells and NK cells [11, 12]. Several studies suggest that CXCR3, together with CXCL10, helps recruit T cells into the airways and lung parenchyma in ALI [2, 13, 14]. Excessive production of IFN-γ and CXCL10 due to the presence of elevated numbers of CD8+ T cells in lung is thought to contribute to chronic obstructive pulmonary disease (COPD) [15]: it appears that CXCL10 production in CD8+ T cells and bronchiolar epithelium triggers CXCR3 to recruit T lymphocytes, contributing to disease symptoms [16].These previous results in ALI and COPD are consistent with our observations of the joint pro-inflammatory effects of CXCR3 and CXCL10 in our mouse model of acute ALI. Taken together, the present and previous findings suggest that the CXCR3-CXCL10 interaction, by recruiting T cells to airways and lung parenchyma, plays a pivotal role in the pathogenesis and progression of ALI. This raises the question of whether lowering CXCR3 expression can slow ALI progression. Indeed, knocking out the CXCR3 gene in mice with experimentally induced acute pancreatitis has been shown to attenuate acute pulmonary injury [17].

The potent anti-inflammatory and immunosuppressive cytokine IL-10, a T-helper-2 (TH2) cytokine, helps control inflammation and modulate adaptive immune responses following tissue damage in numerous inflammatory diseases [18]. IL-10 in the liver modulates the inflammatory response and protects from collateral injury in chronic renal disease, colonitis, and cancer [19, 20]. IL-10 is also up-regulated during certain responses involving tolerant or regulatory T cells. A balance between IL-10 and IFN-γ secreted by TH1 cells is thought to allow an effector response that is sufficiently strong but not excessive as in autoimmunity [20]. IL-10 levels are lower in patients with acute lung inflammation, asthma, COPD, or cystic fibrosis than in healthy individuals, and this deficiency may contribute to pathogenesis [21–23]. Our results demonstrating anti-inflammatory effects of IL-10 in a mouse model of acute ALI are consistent with the literature, and the present work provides the first clues that these effects depend on CXCR3. This extends previous studies suggesting that the anti-inflammatory effects of IL-10 are due in part to its ability to down-regulate the expression of TNF and CXC-family chemokines y. Our findings add weight to the notion that adjusting IL-10 levels may be a useful immunomodulatory therapy in ALI.

Taken together, the results of the present experiments are consistent with a model in which concurrent up-regulation of CXRC3 and down-regulation of IL-10 help drive ALI. A major limitation of the current study is lack of intervention at the suspected molecular pathway and therefore the speculative nature of the conclusion. We appreciate the reviewer for this comment and have added discussion addressing such a limitation in the revised manuscript. Regardless, our findings that the reduction of CD8^+^CD122^+^ regulatory T cells correlated with the increase in CD8^+^ T cells (as well as the changes in IL-10, IFN-γ, and CXCR3) suggest that CD8^+^CXCR3^+^ regulatory T cells could alleviate ALI injury through stimulate the release of IL-10, inhibiting the production of IFN-γ, and inhibiting CXCR3 release. If proven by future studies, this could provide a novel pathway to modulate ALI response.
